# Supplementary material for: DeepGOZero: improving protein function prediction from sequence and zero-shot learning based on ontology axioms
Source: Bioinformatics. 2022 Jun 27;38(Suppl 1):i238–45. doi: 10.1093/bioinformatics/btac256 (PMC9235501; doi:10.1093/bioinformatics/btac256)
Supplement: btac256_Supplementary_Data [file btac256_supplementary_data.pdf]

Supplementary Materials

1 Evaluation metrics

$F_{\max}$ is a maximum protein-centric F-measure computed over all prediction thresholds. First, we compute average precision and recall using the following formulas:

$$pr_i(t) = \frac{\sum_f I(f \in P_i(t) \wedge f \in T_i)}{\sum_f I(f \in P_i(t))} \tag{1}$$

$$rc_i(t) = \frac{\sum_f I(f \in P_i(t) \wedge f \in T_i)}{\sum_f I(f \in T_i)} \tag{2}$$

$$AvgPr(t) = \frac{1}{m(t)} \cdot \sum_{i=1}^{m(t)} pr_i(t) \tag{3}$$

$$AvgRc(t) = \frac{1}{n} \cdot \sum_{i=1}^{n} rc_i(t) \tag{4}$$

where f is a GO class, T_i is a set of true annotations, $P_i(t)$ is a set of predicted annotations for a protein i and threshold t, $m(t)$ is a number of proteins for which we predict at least one class, n is a total number of proteins and I is an indicator function which returns 1 if the condition is true and 0 otherwise. Then, we compute the $F_{\max}$ for prediction thresholds $t \in [0, 1]$ with a step size of 0.01. We count a class as a prediction if its prediction score is greater or equal than t:

$$F_{\max} = \max_t \left\{ \frac{2 \cdot AvgPr(t) \cdot AvgRc(t)}{AvgPr(t) + AvgRc(t)} \right\} \tag{5}$$

$S_{\min}$ computes the semantic distance between real and predicted annotations based on information content of the classes. The information content $IC(c)$ is computed based on the annotation probability of the class c:

$$IC(c) = -log(Pr(c|P(c)) \tag{6}$$

where $P(c)$ is a set of parent classes of the class c. The $S_{\min}$ is computed using the following formulas:

$$S_{\min} = \min_t \sqrt{ru(t)^2 + mi(t)^2} \tag{7}$$

where $ru(t)$ is the average remaining uncertainty and $mi(t)$ is average misinformation:

$$ru(t) = \frac{1}{n} \sum_{i=1}^{n} \sum_{c \in T_i - P_i(t)} IC(c) \tag{8}$$

$$mi(t) = \frac{1}{n} \sum_{i=1}^{n} \sum_{c \in P_i(t) - T_i} IC(c) \tag{9}$$

2 Tables

Ontology	Terms	Proteins	Groups	Training	Validation	Testing
MFO	6,868	43,279	9,337	37,603	2,723	2,953
BPO	21,381	58,729	12,457	50,190	4,513	4,026
CCO	2,832	59,257	13,154	51,311	4,165	3,781

Table 1: Summary of the UniProtKB-SwissProt dataset split by sequence identity of 30%. Similar sequences (>= 30%) were grouped before randomly splitting them into 81/9/10 % split. The table shows the number of GO terms, total number of proteins, number of groups of similar proteins, number of proteins in training, validation and testing sets for the UniProtKB-SwissProt dataset.

**Method**	$F_{\max}$			$S_{\min}$			AUPR			AUC		
	MFO	BPO	CCO	MFO	BPO	CCO	MFO	BPO	CCO	MFO	BPO	CCO
DiamondScore	0.008	0.016	0.048	15.817	46.779	13.750	0.213	0.043	0.128	0.503	0.500	0.501
DeepGOCNN	0.405	0.333	**0.663**	13.373	42.544	**11.006**	0.366	0.273	**0.674**	0.744	**0.680**	**0.760**
MLP	**0.485**	**0.347**	0.620	**12.340**	**41.793**	11.528	**0.454**	**0.301**	0.622	0.676	0.672	0.650
DeepGOZero	0.476	0.343	0.624	12.478	42.168	11.599	0.441	0.285	0.586	**0.766**	0.650	0.601

Table 2: The comparison of performance on the UniProtKB-SwissProt dataset splitted by sequence identity of 30%
.
